# A Meta-Analysis: Intervention Effect of Mind-Body Exercise on Relieving Cancer-Related Fatigue in Breast Cancer Patients

**DOI:** 10.1155/2021/9980940

**Published:** 2021-07-03

**Authors:** Cong Liu, Man Qin, Xinhu Zheng, Rao Chen, Jianghua Zhu

**Affiliations:** ^1^Shanghai University of Sport, Shanghai 200438, China; ^2^Shanghai Lixin University of Accounting and Finance, Shanghai 201209, China; ^3^Donghua Universuty, Shanghai 201620, China

## Abstract

**Objective:**

This paper aims to systematically evaluate the intervention effect of mind-body exercise on cancer-related fatigue in breast cancer patients.

**Methods:**

Databases including PubMed, the Cochrane Library, Embase, Web of Science, CNKI, Wanfang Data, and SINOMED were retrieved to collect randomized controlled trials on the effects of mind-body exercise on relieving cancer-related fatigue in breast cancer patients. The retrieval period started from the founding date of each database to January 6, 2021. Cochrane bias risk assessment tools were used to evaluate the methodological quality assessment of the included literature, and RevMan 5.3 software was used for meta-analyses.

**Results:**

17 pieces of researches in 16 papers were included with a total of 1133 patients. Compared with the control group, mind-body exercise can improve cancer-related fatigue in breast cancer patients. The combined effect size SMD = 0.59, 95% CI was [0.27, 0.92], *p* < 0.00001. Doing Tai Chi for over 40 minutes each time with an exercise cycle of ≤6 weeks can improve cancer-related fatigue in breast cancer patients more significantly. Sensitivity analysis shows that the combined effect results of the meta-analysis were relatively stable.

**Conclusion:**

Mind-body exercise can effectively improve cancer-related fatigue in breast cancer patients.

## 1. Introduction

Breast cancer is not only one of the most common malignant tumors in women around the world but also the main cause of death in women with cancer [1]. Studies have shown that almost in every four cases of female malignant-tumor patients there is one case of a breast cancer patient, and about 520,000 breast cancer patients died in the same period [2]. Cancer-related fatigue (CRF) is a subjective feeling of fatigue associated with cancer or cancer treatment which cannot be alleviated by resting. A large proportion of breast cancer patients experience CRF pain, and their fatigue levels are higher than normal, and higher fatigue levels suggest lower survival rates [3, 4]. At present, there are many treatments for CRF, such as drug treatment, massage, and acupuncture, while as the only recommended intervention by Evidence-based Medicine Group of the Oncology Nursing Society, exercise has been widely recognized in the treatment of CRF [5, 6].

Mind-body exercise (MBE) regards the body, spirit, and outside world as a whole. It is a mental and physical interaction therapy that promotes physical and mental health through asana, pranayama, relaxation, and meditation. The common exercise methods include Tai Chi and Yoga [7]. Studies have shown that mind-body exercise improves cancer-related fatigue in breast cancer patients. Wang et al. [8] conducted a 6-month yoga intervention on breast cancer patients and found that the fatigue of the patients decreased compared with the control group; Linda et al. conducted Tai Chi intervention on breast cancer patients for 12 weeks and the results showed that the fatigue of the patients also decreased [9].

To this end, this study sets to further explore the effect of mind-body exercise on improving cancer-related fatigue in breast cancer patients and to provide evidence for mind-body exercise's function in improving cancer-related fatigue among breast cancer patients.

## 2. Research Methodology

This paper was written in accordance with the requirements of the international meta-analysis writing guidelines (the PRISMA statement for reporting systematic reviews and meta-analyses of studies that evaluate health care interventions: explanation and elaboration). The protocol for this study was registered with INPLASY (202130051).

### 2.1. Inclusion and Exclusion Criteria

#### 2.1.1. Research Design

This is a randomized controlled trial (RCT) of the effect of mind-body exercise on improving cancer-related fatigue in breast cancer patients.

#### 2.1.2. Inclusion Criteria

1. The study was designed as a randomized controlled trial
2. Patients aged 18 years old or above and have been pathologically diagnosed with both breast cancer and CRF were included
3. Intervention therapy was mind-body exercise
4. Chinese and English papers were included

#### 2.1.3. Exclusion Criteria

1. Repeated published studies
2. Studies with inconsistent interventions
3. Studies with unclear or missing outcome indicators

#### 2.1.4. Intervention Measures

1. At least one intervention group used mind-body exercise.
2. If there are multiple data comparisons in the same literature, it would be counted as multiple studies. Based on the conventional drugs and exercise in the experimental group and the control group, the experimental group only added mind-body exercise.

#### 2.1.5. Outcome Indicators

1. The Brief Fatigue Inventory (BFI)
2. European Organization for Research and Treatment Quality of life Questionnaires (EORTC QLQ-C30)
3. Cancer fatigue scale (CFS)
4. Functional Assessment of Chronic Illness Therapy-Fatigue (FACIT-F)
5. Fatigue Symptom Inventory (FSI)
6. Revised Piper Fatigue Scale (PFS-R), Hong Kong Edition

### 2.2. Retrieval Strategy

The computer retrieved the databases including PubMed, Web of Science, the Cochrane Library, Embase, CNKI, Wanfang Data, and SINOMED. The retrieval period started from the founding dates of each database to January 6, 2021. The retrieval strategy adopted the combination of subject words and free words, which was determined after multiple prechecks, supplemented by manual search and the reference 1.3.1 tracking of those papers when necessary. Chinese search terms included the following: mind-body exercise (身心运动), yoga (瑜伽), Tai Chi (太极拳), Qigong (气功), *Baduanjin Qigong* (八段锦), Wuqinxi exercise (五禽戏), Liuzijue Qigong (六字诀), Yijinjing Qigong (易筋经), breast cancer (乳腺癌), cancer-related fatigue (癌因性疲乏), and fatigue (疲乏). The English search terms used the PubMed database as an example:

Mind-Body Therapies [Mesh] OR mind-body therapy [Title/Abstract] OR therapies, mind-body [Title/Abstract] OR mind-body medicine [Title/Abstract] OR mind-body exercise [Title/Abstract] OR Yoga [Mesh] OR Vinyasa [Title/Abstract] OR Tai ji [Mesh] OR Tai-ji [Title/Abstract] OR Tai Chi [Title/Abstract] OR Chi, Tai [Title/Abstract] OR Tai Ji Quan [Title/Abstract] OR Ji Quan, Tai [Title/Abstract] OR Taijiquan [Title/Abstract] OR Qigong [Title/Abstract] OR Ch'i Kung [Title/Abstract] OR baduanjin [Title/Abstract] OR wuqinxi [Title/Abstract] OR yijinjing [Title/Abstract] OR liuzijue [Title/Abstract]
Breast Neoplasms [Mesh] OR Breast Cancer [Title/Abstract] OR Cancer of Breast [Title/Abstract] OR Breast Tumor [Title/Abstract]
Fatigue [Mesh] OR cancer related fatigue [Title/Abstract] OR CRF [Title/Abstract] OR fatigue [Title/Abstract]
(1) AND (2) AND (3)

### 2.3. Literature Screening, Data Extraction, and Quality Evaluation

#### 2.3.1. Literature Screening

Two researchers used independent double-blind methods according to the inclusion and exclusion criteria. The work flow was as follows: read the title and abstract first, conduct a preliminary screening, and then read and download the full text of the documents which meet the criteria; after the screening, the researchers compared the screening results. If there had been a disagreement with the results, a third researcher would have joined in to discuss whether to include the data.

#### 2.3.2. Data Extraction

Two researchers independently extracted the data included in the literature. For materials lacking data or information, they contacted the author via email to obtain and confirm the information. When the included information was inconsistent, they discussed with a third researcher to make a final decision. Data extracted were as follows: (1) basic information (author, year, age, and sample size) and (2) experimental features (movement form, movement cycle, duration, and frequency) and outcome indicators.

#### 2.3.3. Quality Evaluation of Literature

The evaluation criteria of RCT bias risk in the Cochrane Collaboration were used to evaluate the RCT methodological quality in 7 areas: random sequence generation, distribution concealment, blinding of subjects and researchers, blinding of outcome indicator evaluation, complete outcome data, selective reporting, and other biases. The above work was carried out by two researchers independently and each of them cross-checked the work of the other. If there had been a disagreement, a third researcher would join in to jointly decide whether to include it.

#### 2.3.4. Statistical Analysis

The data processing software used was Reviewer Manager 5.3. This meta-analysis strictly followed the PRISMA guidelines. The data extracted in this study were all measurement data, and the main effect parameters were the difference (difference score) between final values after intervention and the measured value at the baseline level; then Formula 1 and Formula 2 were used for calculation.

  Formula 1: *M*=(*M*_1_ − M_2_)
  Formula 2: *S*^2^ = *S*_1_^2^ + *S*_2_^2^ − 2*∗R*∗*S*_1_*∗S*_2_
 
*R* represents constant 0.5

In this study, a number of CRF evaluation indicators were selected. In addition, standardized mean difference (SMD) was selected for analysis to reduce the impact of different measurement methods. The data included in this paper were continuous data, whose confidence interval of effect size was SMD = 95%, and sensitivity analyses were conducted by excluding individual papers one by one. *P* value and *I*^2^ were adopted for heterogeneity test. If the study results showed statistical heterogeneity (*I*^2^ ≥ 50%, *P* < 0.10), the random-effects model would be used; otherwise, the fixed-effects model would be used.

## 3. Results

### 3.1. Literature Retrieval Results

**Figure 1:**
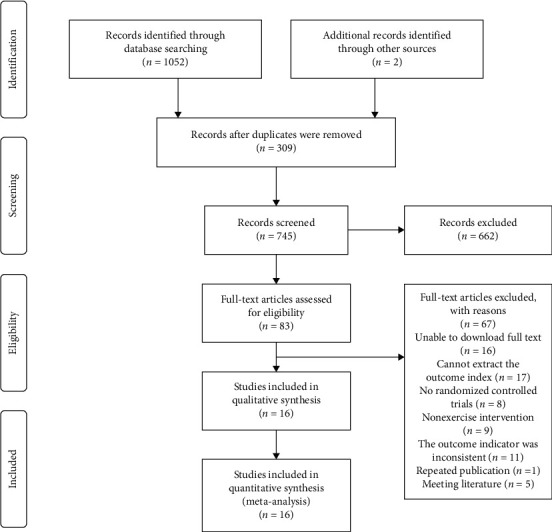
Literature screening process.

Through the retrieval of databases in PubMed, the Cochrane Library, Embase, Web of Science, CNKI, Wanfang, and SINOMED, a total of 1052 papers were retrieved, and 2 other papers were supplemented by tracking other resources. After deduplication, reading the title and abstract, rescreening the full text, and excluding unqualified documents, 16 documents were finally obtained, as shown in Figure 1.

### 3.2. Basic Characteristics of the Included Literature

This study included 17 pieces of research and 1133 participants in 16 documents [8, 10–24]. There were 3 Chinese papers and 13 English papers. The publication period ranged from 2007 to 2019, as shown in Table 1.

### 3.3. Features of the Included Literature Intervention

The exercise forms involved in the included literature included yoga, Tai Chi, and Qigong. The most common exercise type was yoga; the exercise lasted from 40 to 90 minutes, and the time lasting for over 40 minutes was the majority; the exercise cycle was mostly more than 6 weeks; the frequency was mostly more than 3 times a week. Five studies [14, 19–22] selected BFI as the outcome indicator; three studies [11, 13, 16] selected EORTC QLQ C30 as the outcome indicator; three studies [15, 17, 18] selected FACIT-F as the outcome indicator; three studies [8, 10] selected CFS as the outcome indicator; two studies [12, 23] selected FSI as the outcome indicator; and one study [24] selected PFS-R as the outcome indicator. The detailed outcome indicators were shown in Table 1.

### 3.4. Quality Evaluation of the Included Literature

**Figure 2:**
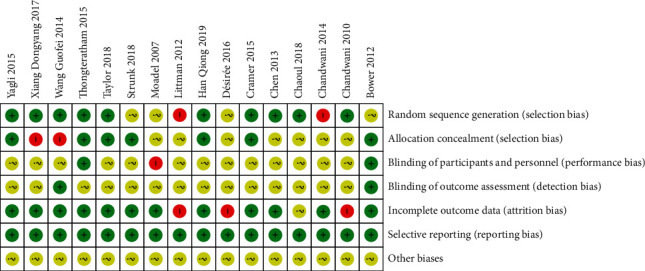
Assessment of bias risk.

All the included literature were randomized controlled trials, among which ten papers described the generation of randomization [8, 10–12, 14, 18–21, 24]; seven papers wrote the method of random allocation concealment [11–14, 18, 23, 24]; fourteen papers did not describe whether the researchers and subjects were blinded; two papers blinded the result evaluation [8, 23]; twelve papers had complete result report, of which three did not report the reasons for the number of missing subjects [16, 17, 21], as shown in Figure 2.

### 3.5. Results of the Meta-Analyses

#### 3.5.1. Results of the Effect of Mind-Body Exercise on CRF in Breast Cancer Patients

**Figure 3:**
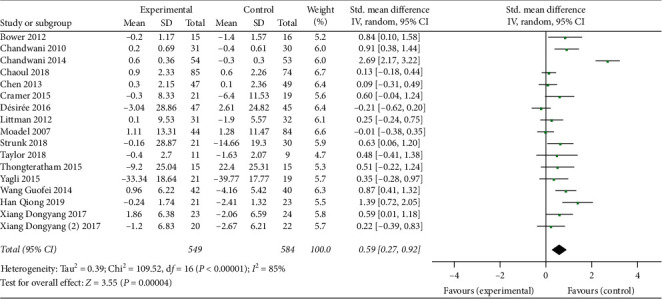
Forest plots of CRF effects of mind-body exercise on breast cancer.

A total of 17 researches in 16 papers were analyzed to compare the differences of cancer-related fatigue between the mind-body exercise group and the control group of 1133 breast cancer patients. As shown in Figure 3, the heterogeneity test results showed that *I*^2^ = 85% and *P* ≤ 0.0001, indicating that the studies had relatively high heterogeneity, so the random-effects model was selected for analysis. The results of the meta-analysis showed that the combined effect size SMD = 0.59, 95% CI was [0.27, 0.92], and *P*=0.0004, indicating that, compared with the control group, the mind-body exercise group could better reduce the fatigue of breast cancer patients.

#### 3.5.2. A Subgroup Analysis of the Effect of Mind-Body Exercise on CRF of Breast Cancer Patients

To explore potential sources of heterogeneity, subgroup analyses of potential sports variables were performed (Table 2).

The subgroup analyses were performed according to different exercise types, which consisted of Tai Chi, yoga, and other types. The SMD in the other types' group was 0.32, 95% CI (−0.21, 0.84), *P* > 0.05, indicating that the other types' group had no significant difference in the improvement of CRF in breast cancer patients compared with the control group. The SMD in the Tai Chi group was 0.96, 95% CI (0.10, 1.82), *P* < 0.05, whereas the SMD in the yoga group was 0.59, 95% CI (0.18, 0.99), *P* < 0.05, which indicated that Tai Chi had a better effect on the improvement of CRF in breast cancer patients.

This subgroup analysis was performed according to various exercise cycles, which consisted of ≤6 weeks and >6 weeks. Seven studies lasted for ≤6 weeks, with heterogeneity among studies: *I*^2^ = 92% and *P* < 0.00001. Ten studies lasted for >6 weeks with heterogeneity among studies: *I*^2^ = 69% and *P*=0.007. The *P* values of SMD in both groups were all less than 0.05, indicating that the CRF of both two groups of breast cancer patients was improved compared with the control group. The SMD of total effect size of exercise cycle lasting for more than 6 weeks was 0.50, while the SMD of total effect size of the exercise cycle lasting for less than 6 weeks was 0.71, indicating that the exercise cycle lasting for ≤6 weeks better improved the CRF of breast cancer patients.

Another subgroup analysis was performed according to different exercise duration, which could be divided into ≤40 minutes and >40 minutes. Three studies lasted for ≤40 minutes, but the SMD = 0.24, 95% CI (−0.05, 0.53), *P*=0.10. It indicated that there was no significant difference in the improvement of CRF in breast cancer patients who exercised for less than 40 minutes compared with the control group. However, in the group that exercised for more than 40 minutes, SMD = 0.66, 95% CI (0.27, 1.06), *P* < 0.01, indicating that, with the exercise time lasting for more than 40 minutes, the improvement of CRF in breast cancer patients is significantly better than that of the control group.

#### 3.5.3. Sensitivity Analysis of the Effect of Mind-Body Exercise on CRF in Patients with Breast Cancer

In order to explore whether the heterogeneity of the studies was caused by individual studies, this study conducted a sensitivity analysis of the studies of heterogeneous mind-body exercise on CRF in breast cancer patients and analyzed the combined effect size by excluding individual studies one by one (see Table 3).

All the mind-body exercise studies in Table 3 were gathered to calculate the combined effect size of the CRF in breast cancer patients: SMD = 0.59, 95% CI (0.27, 0.92), *P*=0.0004. After excluding the data of Chandwani et al. [22], the combined effect size was SMD = 0.43, 95 CI (0.22, 0.64), *P* < 0.00001, *I*^2^ = 60%, with a reduced heterogeneity. After excluding other individual studies, the combined effect size was SMD between 0.43 and 0.65, the range of *I*^2^ was 85%–86%, *P* value was less than 0.0001, and the heterogeneity was changed only slightly.

## 4. Discussion and Analysis

The results of this study suggest that mind-body exercise can improve CRF in breast cancer patients. This may have to do with the two following respects. First, mind-body exercise can improve the CRF of breast cancer patients by regulating the levels of IL-6, TNF-*α*, and IL-1*β*. Wood et al. [25] believed that CRF symptoms may be caused by the increase of proinflammatory factors tumor necrosis factor-*α* (TNF-*α*) and interleukin-1*β* (IL-1*β*). Interleukin-6 (IL-6) could inhibit proinflammatory factors such as TNF-*α* and IL-1*β*, leaving the body in an environment of anti-inflammatory factors [26]. Meanwhile physical exercise could make skeletal muscle produce a large amount of IL-6 and increase the IL-6 concentration in serum [27]. The reason why mind-body exercise improved the CRF of breast cancer patients may be that which meant mind-body exercise promoted skeletal muscle to produce a large amount of IL-6 as well as inhibiting TNF-*α* and IL-1*β*, thereby reducing the CRF in breast cancer patients. An animal study also reached the same conclusion. He et al. [28] conducted a 6-week swimming exercise intervention on a CRF model of rat breast cancer and found that moderate-intensity and low-intensity aerobic exercise could alleviate the rat's CRF, increase IL-6, and reduce the levels of TNF-*α* and IL-1*β*. Second, the improvement of CRF by mind-body exercise may be related to the reduction of mitochondrial injury and protection of mitochondria. Mitochondria are important sites for respiration and energy metabolism [29]. Mitochondrial dynamics are involved in processes such as ATP synthesis, oxidative stress, and apoptosis and are associated with breast cancer invasion and metastasis [30]. An animal study found that the mitochondrial membrane potential (MMP) in the skeletal muscle of the CRF model of rat breast cancer was significantly reduced, the synthesis of adenosine triphosphate (ATP) and glutathione (GSH) was reduced, and the synthesis of malondialdehyde (MDA) increased. It indicated that the cause of CRF may be related to the mitochondrial oxidative damage and mitochondrial dysfunction. After aerobic exercise intervention in rats, it was found that CRF symptoms were alleviated. Moreover, MMP and synthesis of ATP and GSH increased, and MDA decreased significantly. It was speculated that the alleviation of the fatigue symptoms was related to the reduction of skeletal muscle mitochondrial oxidative damage and protection of mitochondrial function [31].

The results of this study showed that mind-body exercise can improve CRF in breast cancer patients. Previous studies have also obtained similar results. Carson et al. [32] conducted an 8-week yoga intervention in breast cancer patients and found that their fatigue levels were reduced; Galantino et al. [33] performed a Tai Chi intervention for 60 minutes each time, twice a week for 8 weeks in breast cancer patients, and found that their fatigue symptoms improved. The subgroup results showed that doing Tai Chi for more than 40 min had a better effect on CRF improvement among breast cancer patients. A previous meta-analysis showed that Qigong and Tai Chi had a positive effect on relieving fatigue of breast cancer patients [34]; Larkey et al. [9] conducted Tai Chi intervention in breast cancer patients for 12 weeks and found that their persistent fatigue symptoms relieved; Cao Xin [35] et al. conducted mindfulness training interventions in breast cancer patients and found that the CRF levels of the observation group during the fourth chemotherapy and the sixth chemotherapy were both lower than those of the second chemotherapy. Jin Cuifeng et al. [36] also found that the longer intervention, the better the intervention effect of yoga on cancer-related fatigue of breast cancer patients. Wang et al. [37] conducted yoga interventions on breast cancer patients and found that, compared with the conventional control group, the improvement of fatigue symptoms of the yoga group was better, and the duration caused significant fatigue symptom difference between the two groups. Vadiraja et al. [38] conducted a 60-minute yoga intervention per day on 44 breast cancer patients and found that, compared with the control group, the fatigue degree of the intervention group was significantly reduced over time. The intervention effect was better when the exercise cycle was ≤6 weeks. This result is different from previous studies of others, which might have to do with the limited number of included literature and different evaluation indexes.

## 5. Limitations

The literature included in this study has some limitations; there is a certain lack of description about the differences in the courses of breast cancer patients and the differences among individual patients; the study used multiple scales, and there were also differences in scoring standards.

## 6. Conclusions

Mind-body exercise can improve the CRF in breast cancer patients. Doing Tai Chi for ＞40 minutes each time with an exercise cycle of ≤6 weeks has a better effect on relieving CRF in breast cancer patients. Sensitivity analysis shows that the results of this study are relatively stable. However, due to the implications of various factors, more standardized and higher-quality researches should be completed in the future.

## Figures and Tables

**Table 1:** Basic characteristics of the included researches.

Included literature	Age (C/T)	Sample size (C/T)	Type of exercise	Exercise duration (min)	Exercise cycle (weeks)	Exercise frequency (per week)	Outcome indicators
Chandwani et al. [22]	52.11/52.38	54/53	Yoga	60	6	3	①
Chandwani et al. [21]	54.02/51.39	31/30	Yoga	60	6	2	①
Chaoul et al. [20]	49/49.5	85/74	Yoga	75–90	6	≥2	①
Desiree et al. [16]	51.4/51	47/45	Yoga	60	12	-	②
Chen et al. [19]	44.7/45.3	47/49	Qigong	40	5∼6	5	①
Littman et al. [17]	58.2/60.6	31/32	Yoga	65–85	24	5	③
Moadel et al. [15]	54.23/55.11	44/84	Yoga	90	24	≥1	③
Taylor et al. [14]	52.6/54.9	15/18	Yoga	75	8	1	①
Strunk et al. [13]	51.5/54.2	21/30	Kyusho Jitsu	90	24	2	②
Yagli et al. [11]	47.38/49.89	28/24	Aerobics + yoga	60	6	3	②
Cramer et al. [18]	50/48.3	21/19	Yoga	90	12	1	③
Xiang et al. [10]	≥18	23/24	Yoga	40	4	3∼4	④
Xiang et al. [10]	≥18	20/22	Yoga + music	40	4	3∼4	④
Wang et al. [8]	18∼60	42/40	Yoga	50	16	4	④
Bower [23]	53.3/54.4	15/16	Yoga	90	12	2	⑤
Thongteratham et al. [12]	-	15/15	Taiji Qigong	60	12	3	⑤
Han et al. [24]	45.52/46.39	23/21	Eight-type taijiquan	＞40	12	5	⑥

*Note*. C: control group; *T*: experimental group; “-”: unspecified information. ① BFI; ② EORTC QLQ C30; ③ FACIT-F; ④ CFS; ⑤ FSI; ⑥ PFS-R.

**Table 2:** A subgroup analysis of sports variables on the effect of CRF intervention in patients with breast cancer.

Research on intervention characteristics	Number of experiments	SMD (95% CI)	*I* ^2^ (%)	*P* value	*P* (SMD)
*Exercise type*					
Tai Chi	2	0.96 [0.10, 1.82]	67	0.08	0.03
Yoga	13	0.59 [0.18, 0.99]	88	0.004	＜0.0001
Other types	2	0.32 [−0.21, 0.84]	57	0.13	0.23
Total amount	17	0.59 [0.27, 0.92]	85	＜0.00001	0.0004

*Exercise cycle* (*weeks*)					
≤6	7	0.71 [0.04, 1.37]	92	0.10	＜0.00001
＞6	10	0.50 [0.18, 0.81]	69	0.0007	0.002

*Exercise duration* (*min*)					
≤40	3	0.24 [−0.05, 0.53]	0	0.37	0.10
＞40	14	0.66 [0.27, 1.06]	88	＜0.00001	0.0004

Total amount	17	0.59 [0.27, 0.92]	85	＜0.00001	0.0004

**Table 3:** Combined effect of CRF after excluding individual studies.

Elimination of literature	SMD	95% CI	*P* (merger effect)	*I* ^2^ (%)	*P*
Chandwani et al. [21]	0.58	0.23, 0.92	0.001	86	＜0.00001
Chandwani et al. [22]	0.43	0.22, 0.64	＜0.00001	60	0.001
Chaoul et al. [20]	0.63	0.27, 0.99	0.0006	86	＜0.00001
Chen et al. [19]	0.63	0.28, 0.98	0.0004	86	＜0.00001
Cramer et al. [18]	0.60	0.25, 0.94	0.0007	86	＜0.00001
Desiree et al. [16]	0.65	0.31, 0.99	0.0002	85	＜0.00001
Littman et al. [17]	0.62	0.27, 0.97	0.0005	86	＜0.00001
Moadel et al. [15]	0.64	0.29, 0.99	0.0003	85	＜0.00001
Strunk et al. [13]	0.59	0.25, 0.94	0.0008	86	＜0.00001
Taylor et al. [14]	0.60	0.26, 0.94	0.0006	86	＜0.00001
Yagli et al. [11]	0.61	0.26, 0.96	0.0005	86	＜0.00001
Wang et al. [8]	0.63	0.27, 1.00	0.0006	85	＜0.00001
Xiang et al. [10]	0.65	0.30, 1.01	0.0003	86	＜0.00001
Xiang et al.(2) [10]	0.68	0.32, 1.03	0.0002	86	＜0.00001
Bower et al. [23]	0.58	0.24, 0.92	0.0009	86	＜0.00001
Thongteratham et al. [12]	0.60	0.26, 0.94	0.0006	86	＜0.00001
Han et al. [24]	0.60	0.26, 0.95	0.0006	85	＜0.00001

## Data Availability

The raw data supporting this manuscript are from previously reported studies and datasets, which have been cited. The processed data are available in the supplementary files.

## Supplementary Materials

Supplementary Materials The processed data of the paper.
