# Supplementary material for: A Meta-Analysis: Intervention Effect of Mind-Body Exercise on Relieving Cancer-Related Fatigue in Breast Cancer Patients
Source: Evid Based Complement Alternat Med. 2021 Jul 3;2021:9980940. doi: 10.1155/2021/9980940 (PMC8275388; doi:10.1155/2021/9980940)
Supplement: Supplementary Materials — The processed data of the paper. [file 9980940.f1.docx]

|  | E | | | C | | |
| --- | --- | --- | --- | --- | --- | --- |
| 纳入研究 | M | SD | N | M | SD | N |
| Bower2012 | -0.2 | 1.17 | 15 | -1.4 | 1.57 | 16 |
| Chandwani2010 | 0.2 | 0.69 | 31 | -0.4 | 0.61 | 30 |
| Chandwani2014 | 0.6 | 0.36 | 54 | -0.3 | 0.3 | 53 |
| Chaoul2018 | 0.9 | 2.33 | 85 | 0.6 | 2.26 | 74 |
| Chen 2013 | 0.3 | 2.15 | 47 | 0.1 | 2.36 | 49 |
| Cramer2015 | -0.3 | 8.33 | 21 | -6.4 | 11.53 | 19 |
| Désirée2016 | -3.04 | 28.86 | 47 | 2.61 | 24.82 | 45 |
| Littman2012 | 0.1 | 9.53 | 31 | -1.9 | 5.57 | 32 |
| Moadel2007 | 1.11 | 13.31 | 44 | 1.28 | 11.47 | 84 |
| Strunk 2018 | -0.16 | 26.87 | 21 | -14.66 | 19.3 | 30 |
| Taylor 2018 | -0.4 | 2.7 | 11 | -1.63 | 2.07 | 9 |
| Thongteratham2015 | -9.2 | 25.04 | 15 | -22.4 | 25.31 | 15 |
| Yagli2015 | -33.34 | 18.64 | 21 | -39.77 | 17.77 | 19 |
| 王国妃（WANG Guofei）2014 | 0.96 | 6.22 | 42 | -4.16 | 5.42 | 40 |
| 韩琼(HAN Qiong)2019 | -0.24 | 1.74 | 21 | -2.41 | 1.32 | 23 |
| 项东阳(XIANG Dongyang)2017 | 1.86 | 6.38 | 23 | -2.06 | 6.59 | 24 |
| 项东阳（2）(XIANG Dongyang)2017 | -1.2 | 6.83 | 20 | -2.67 | 6.21 | 22 |
